# A Validated HPLC-PDA-HRMS Method to Investigate the Biological Stability and Metabolism of Antiparasitic Triterpenic Esters

**DOI:** 10.3390/molecules26237154

**Published:** 2021-11-26

**Authors:** Laura Schioppa, Fanta Fall, Sergio Ortiz, Jacques H. Poupaert, Joelle Quetin-Leclercq

**Affiliations:** 1 Pharmacognosy Laboratory, Louvain Drug Research Institute (LDRI), Université Catholique de Louvain (UCLouvain), Avenue E. Mounier, B1 72.03, B-1200 Brussels, Belgium; fanta.fall@uclouvain.be (F.F.); sergio.ortiz@uclouvain.be (S.O.); joelle.leclercq@uclouvain.be (J.Q.-L.); 2 Medicinal Chemistry Research Group (CMFA), Louvain Drug Research Institute (LDRI), Université Catholique de Louvain (UCLouvain), Avenue E. Mounier, B1 72.04, B-1200 Brussels, Belgium; jacques.poupaert@uclouvain.be

**Keywords:** triterpenes, stability, HPLC-PDA/MS, in vitro stability, metabolomics, parasitic infections

## Abstract

Pentacyclic triterpenes (PTs) are commonly found in medicinal plants with well-known antiparasitic effects. Previous research on C-3 and C-27 triterpenic esters showed effective and selective in vitro antiparasitic activities and in vivo effectiveness by parenteral routes. The aim of this study was to determine triterpenic esters’ stability in different biological-like media and the main microsomal degradation products. An HPLC-PDA method was developed and validated to simultaneously analyze and quantify bioactive triterpenic esters in methanol (LOQ: 2.5 and 1.25–100 µg/mL) and plasma (LOQ: 5–125 µg/mL). Overall, both triterpenic esters showed a stable profile in aqueous and buffered solutions as well as in entire plasma, suggesting gaining access to the ester function is difficult for plasma enzymes. Conversely, after 1 h, 30% esters degradation in acidic media was observed with potential different hydrolysis mechanisms. C-3 (15 and 150 µM) and C-27 esters (150 µM) showed a relatively low hepatic microsomal metabolism (<23%) after 1 h, which was significantly higher in the lowest concentration of C-27 esters (15 µM) (>40% degradation). Metabolic HPLC-PDA-HRMS studies suggested hydrolysis, hydroxylation, dehydration, *O*-methylation, hydroxylation and/or the reduction of hydrolyzed derivatives, depending on the concentration and the position of the ester link. Further permeability and absorption studies are required to better define triterpenic esters pharmacokinetic and specific formulations designed to increase their oral bioavailability.

## 1. Introduction

Neglected tropical diseases (NTDs) account for 1% of the global disease burden, and more than 1.7 billion people require treatment for at least one NTD every year. For the fifth consecutive year, NTDs threatened more than one billion people despite the global implementation after 2008 [1]. Among protozoan infections, Human African Trypanosomiasis (HAT) caused by *Trypanosoma brucei* parasites still remains present in over 20 countries. In spite of registered cases dropping in 2015, an under-detection of cases should be considered during the reported incidence assessment [2]. Malaria, caused by *Plasmodium* protozoans, is the most widespread parasitic infection, with 229 million reported cases in 2019. A scale-up intervention series over the last decade helped to cut the mortality rate by 44% between 2010 and 2019 [3]. Even with the latest research and development improvement, the insufficient economic returns for developing new NTD drugs have strongly limited pharmaceutical industry R&D, and so far, most infected people cannot afford for treatments even when they are available [4]. In this context and facing available treatment limitations as reduced efficacy, increased parasite resistance [5] and to aim to a potential large-scale distribution, new innovative oral therapies are crucially needed for such disease control or potential long-term elimination. Among promising natural compounds [6], triterpenoids are ubiquitous phytochemicals prevalent in the plant kingdom with significant pharmacological activities [7,8]. A potential treatment with triterpenes could allow for a multifactorial approach for targeting parasite growth and infection-induced symptoms for optimal disease control and resistance-development prevention. A mixture of eight coumaroyl/feruloyl triterpenic esters (8TTE) with selective in vitro and i.p. in vivo antimalarial activity was identified in *Keetia leucantha* twigs, supporting their traditional use and triterpenes interest in parasitology [9,10]. Moreover, recent hemi-synthesis of ursane C-3 triterpenic esters highlighted the in vitro and in vivo potential against *Trypanosoma brucei* of ursolic acid-3-*O*-phenyl propionate (UA-3-*O*PP) [11]. The present study aimed to investigate the stability of these promising antiparasitic triterpenic esters and their potential to target malaria and HAT infections. This is the first study on the biological stability and metabolism of these triterpenic esters in different physiological conditions to obtain crucial information to assess pharmacokinetic profiles and evaluate their potential for oral formulation and administration. To evaluate this stability, an HPLC-PDA-MS method was firstly developed and validated.

## 2. Results and Discussion

### 2.1. Method Validation

#### 2.1.1. Selectivity

Selectivity and peak purity were assessed by retention times comparison and mass spectra analysis with reference standards (Supplementary Materials Figure S1). Three retention times corresponding to the beginning, the middle and the end of each peak were analyzed by HPLC-PDA-MS in ESI (-) to assess the molecular mass and spectra along the peak(s) (Supplementary Materials Figure S2). Molecular ion peaks at *m*/*z* 587.41477 corresponded to [M − 1]^−^ of UA-3-*O*-PP and *m*/*z* 617.39047 or 647.4013 to [M − 1]^−^ of coumaric or ferulic 8TTE derivatives, respectively. Method selectivity for plasma samples was assessed by HPLC-PDA showing no interfering peak at the 8TTE (310 nm), IS (281 nm) and UA-3-*O*-PP (210 nm) retention times in blank plasma.

#### 2.1.2. Response Function

To demonstrate quantification reliability in the methanol and plasma, different regression models from calibration standards were analyzed [12]. For methanolic samples, linear regression was selected as the most adequate one, with 95% expectation tolerance intervals included inside the ±20 % acceptance limits for each concentration level of the validation standards (except the lowest one). For plasma samples, a square root transformation was performed to obtain the best calibration curve model. This total error concept simplifies decision making and reduces risks associated with procedure use [13].

#### 2.1.3. Trueness, Precision and Accuracy

Trueness was calculated at each concentration level of the validation standards and expressed in relative bias (RB) [14,15]. For methanolic samples, the calculated relative bias for the 8TTE mixture and UA-3-*O*-PP were less than 7.24% and 5.05%, respectively, showing the good trueness of the method (Table 1). Precision was evaluated intra-day (repeatability) and inter-day (intermediate precision) and expressed as relative standard deviations (RSD) [13,16]. Methanolic samples deviations were 4.78 and 2.18% maximum for repeatability and 4.41 and 3.84% for the intermediate precision for 8TTE mixture and UA-3-*O*-PP, respectively (Table 1). Concerning plasma samples (Table 2), the relative bias of the 8TTE mixture and UA-3-*O*-PP were no more than 15.47 and 13.03%, respectively. The repeatability was less than 2.66 and 2.89%, and the intermediate precision values were 2.49 and 3.25% for 8TTE mixture and UA-3-*O*-PP, respectively (Table 2). Repeatability and intermediate precision data show the excellent precision of the method for both compounds. All the trueness and precision results follow EMA guidelines criteria (≤15%) [17]. Accuracy profiles, evaluating the sum of systematic and random errors (total error) [12,14,15], indicated the relative upper and lower 95% β-expectation tolerance limits were inside the acceptance limits set at ±20% (Supplementary Materials Figure S3). All the validation results are presented in Table 1 and Table 2.

#### 2.1.4. Detection and Quantification Limits

The limit of detection (LOD) is estimated for 8TTE mixture and UA-3-*O*-PP as 0.38 and 0.75 µg/mL in methanol and 1.5 µg/mL in plasma matrices, respectively, by the signal/noise method from the European Pharmacopoeia [18]. The limit of quantification was determined as the smallest tested concentration of the 95% β-expectation tolerance limits remaining inside the ±20% acceptance limits. It was then set at 1.25 µg/mL for 8TTE or 2.5 µg/mL for UA-3-*O*-PP in methanol and 5 µg/mL for both in plasma. Considering the two-fold concentration of plasma sample during treatment procedure, the method allows a precise quantification and detection in plasma from 2.5 µg/mL.

#### 2.1.5. Uncertainty of Measurement

To characterize values dispersion during routine analysis, the uncertainty of measurement was determined [19,20]. The expanded uncertainty is calculated by applying a coverage factor of k = 2 (corresponding to a 95 % confidence interval around the estimated result). The maximum relative expanded uncertainty of the mixture of 8TTE is 9.8% while for UA-3-*O*-PP is 8.46%, both inside the ±20% acceptance limits. All estimated values for each validation standard concentration level are summarized in Table 3.

#### 2.1.6. Linearity

To determine the linearity, the fitness of a regression line between back-calculated concentrations and exact concentrations was evaluated [20,21]. The estimated concentrations were plotted as a function of the introduced concentrations, and the built regression line was compared to the identity line y = x. For both methanolic and plasma samples, calculated slope values were close to 1, supporting the good linearity of the method alongside the absolute 95 % β-expectation tolerance limits within the absolute acceptance limits (±20%) (Supplementary Materials Figure S4).

### 2.2. Stability Profiles Investigation

**Figure 1:**
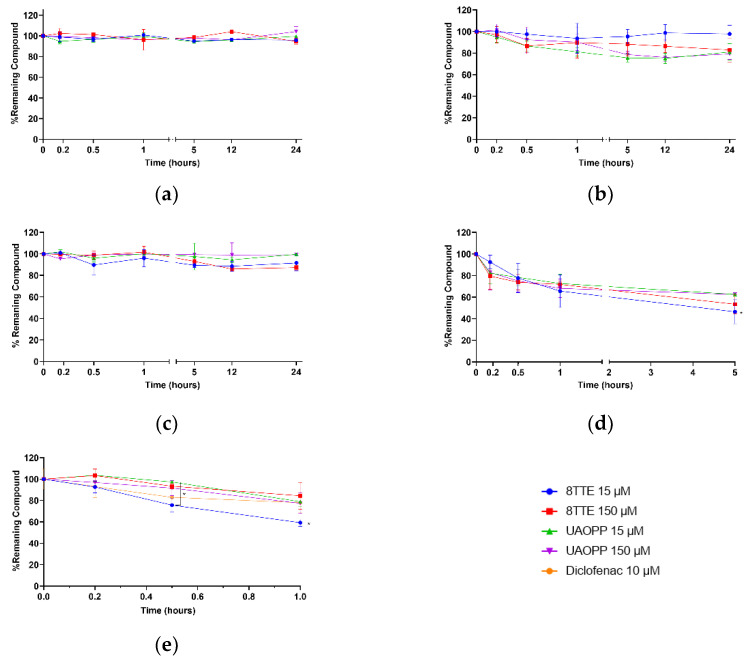
8TTE, UA-3-*O*-PP and positive controls stability profiles in different simulated physiologic conditions at two different concentrations (15 µM–150 µM): (**a**) water; (**b**) phosphate Buffer 0.1 M pH 7.4; (**c**) entire plasma; (**d**) HCl pH 1.2; (**e**) pooled male mouse microsomes. Non-parametric ANOVA analysis: *p* < 0.05 (Kruskal–Wallis and Dunn’s tests).

Determination of the stability of new chemical entities is typically investigated in drug discovery or lead optimization [22]. The particular interest in the tested molecules is due to their aromatic ester functional groups in key positions, which induces increased antiplasmodial activity (natural esters) or decreases cytotoxicity (synthetic ester) compared to the corresponding triterpenic acids [9,23]. Furthermore, esterification can also lead to a bioavailability improvement, with a better potential for oral administration [24], a golden standard in the contest of tropical parasitic infections treatments. A stability profile investigation on these molecules was carried out in different environments and physiological conditions so as to mimic the pH environments of blood, stomach and intestine. A simple model-independent method was used, in which the percentage of remaining compound over time was determined by analyte and internal standard signals ratio and normalized to the value obtained at t = 0. Results of the stability profiles are presented in Figure 1. All tested compounds, in aqueous and buffered conditions as well as entire plasma, were stable at both concentrations for 24 h (loss < 20%, Figure 1a–c).

**Figure 2:**
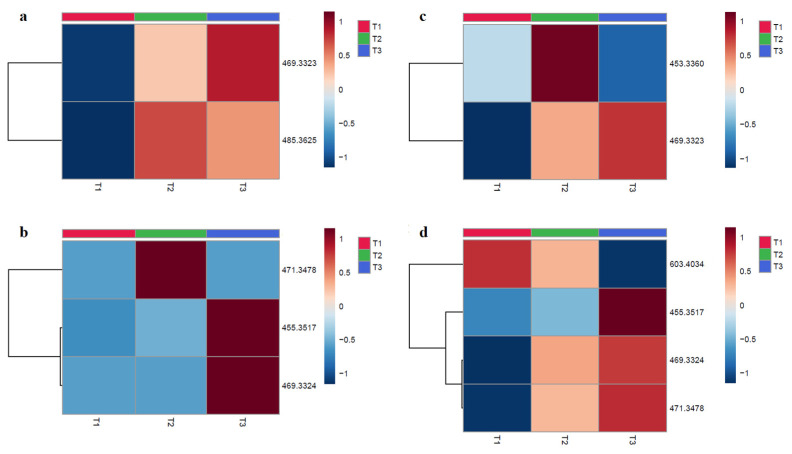
Heatmaps of molecular ions intensity in function of the tested timepoints for 8TTE and UA-3-*O*-PP. Each column represents an average of three samples (n = 3), and the rows represent a corresponding *m/z*. (red color = more expressed; blue color = less expressed): (**a**) 8TTE 15 µM; (**b**) 8TTE = 150 µM; (**c**) UA-3-*O*-PP 15 µM; (**d**) UA-3-*O*-PP 150 µM (T1 = 0 min, T2 = 30 min; T3 = 60 min). Analysis was performed using Euclidian distance method with ward clustering algorithm after normalization to sample median and Pareto scaling.

These results clearly indicate that near neutrality of the aqueous medium, the ester function remains acceptably stable, even for extended periods, showing that esters should be stable in fresh aqueous solutions but also in the intestine after administration. It is well established that ester hydrolysis is catalyzed by both acids and bases. In acidic medium, some degradation is actually observed (37–54% degradation after 5 h). Indeed, higher susceptibility to acidic medium (pH 1.2) was exhibited for both esters with the natural ester’s mixture being less stable after 5 h and significantly less stable for the lowest concentration (53.5% degradation) (Figure 1d). At this pH, the different stability of the C-3 and C-27 triterpenic esters could be explained by different underlying hydrolysis mechanisms. While for the latter, a classic acid catalyzed ester hydrolysis (oxygen protonation followed by nucleophilic attack) applies, a potential SN1-like process might take place for C-3 esters. More in detail, when the carbonyl of the C-3 ester is protonated, the back strain produced by the neighboring gem-dimethyl group at the C-4 position may induce the expulsion of the whole ester group by breaking the C(3)-*O* bond and therefore forming a carbocation which subsequently recombines with a water molecule to regenerate ursolic acid. This process is specially facilitated when the C(3)-*O* bond is longer than it would be in a less crowded ester. Further research is necessary to confirm this hypothesis. However, to prevent stomach degradation, different formulation approaches can be considered to improve their stability and bioavailability, e.g., gastro-resistant coating, cyclodextrin complexation, liposomes, nanoemulsions [25,26]. This study also aimed to evaluate stability and primary biotransformation(s) occurring in plasma and liver as they are linked to drug excretion and detoxification. According to literature, triterpenic acids as ursolic, oleanolic, asiatic and betulinic acids, are bound to plasma proteins for more than 95%, leading to a low clearance and modifications of tertiary/secondary plasma proteins structures [27,28,29]. The obtained plasma results on triterpenic esters (Figure 2c) may also indicate a similar interaction inducing a low degradation rate and clearance. Indeed, the present plasma data did not show any behavioral difference related to the ester position on the triterpenic skeleton (C-3 or C-27). The metabolic (in)stability of the esters following incubation with mouse liver microsomes (Figure 2e) was also investigated. The degradation was significantly high for 8TTE (>40% after 1 h) at 15 µM, while it was more limited for the 3 other samples (max 22% after 1 h). It was observed a more stable behavior in C-3 esters compared to C-27 ones and a possible saturation for C-27 esters, but also a general slow metabolism in mouse CYP 450 enzymes. In vitro half-lives of 76.8 min and 264.3 min for 8TTE and UA-3-*O*-PP, respectively, were calculated. This is higher than non-esterified triterpenic acids as oleanolic acid, which showed a half-life between t_1/2_ 41.9 and 52.7 min [28]. T_1/2_ is a common parameter in drugs screening, as it does not require metabolites identification but it presents some limitations as a first-order kinetic hypothesis. Current data allowed an in vitro intrinsic clearance (C_intr_) estimation assuming a linear kinetic. The in vitro estimated pharmacokinetic parameters are tabulated in Table 4. Improvement in half-life and pharmacokinetic parameters through a 3-*O*-esterification of oleanolic acid (3β-ester) was previously shown, which is in accordance with the presented findings [30]. The data obtained for tested esters are indicative of a medium and low mouse in vitro intrinsic clearance (µL/min/mg protein) for C-27 and C-3 esters, respectively, while the reference compound, diclofenac, also showed a low C_intr_ (9 µL/min/mg), yet this was higher than previously reported (5 µL/min/mg) [31]. Medium to low clearance drugs are more likely to be more bioavailable and better candidates for oral treatment compared to highly cleared compounds characterized by a shorter duration of action. As these in vitro intrinsic clearance’s estimations do not consider constituents bound to microsomes medium, they are indicative but not sufficient to unambiguously evaluate a drug’s clearance.

### 2.3. Metabolites Analysis

The metabolites analysis of tested compounds was performed after contact with male mouse pooled microsomes at two concentrations (15 µM and 150 µM). LC-PDA-HRMS analyses of treated compounds were made after different incubation times (T1 = 0 min, T2 = 30 min, T3 = 60 min) at both concentrations and showed a variation over time and in concentration. Potential metabolites are tabulated in Table 5. The metabolites structures are proposed with an identification level of 2. The ions annotation in metabolomics was conducted at different levels; level 1 corresponds to the strongest degree of identification (two orthogonal analytical techniques applied with a chemical reference standard); level 2 is putatively annotated compounds (with public or commercial libraries); level 3 is putatively characterized compound classes; and level 4 corresponds to molecules without identification [32]. The proposed metabolites of the hemisynthetic ester UA-3-*O*-PP were potentially obtained by various phase-1 metabolic transformations, such as hydrolysis (giving ursolic acid) and hydroxylation, with or without reduction in the pentacyclic skeleton. For the 8TTE mixture, we observed, in addition to hydrolysis, an aglycone dehydration, as was already observed for polyhydroxylated triterpenic acids such as maslinic, asiatic and madecassic acids [33,34] and a reduction or a methylation of this aglycone. To confirm the proposed metabolites formation, further analysis is required with comparison to standards. The heatmaps represented in Figure 2 were obtained after normalization to sample median and display the intensity modification (formation or degradation) of the molecular ions of the proposed metabolites from HPLC-PDA-HRMS data. For the 8TTE mixture, hydrolysis with aglycone reduction (*m*/*z* 469.3323) appeared to increase with time, while aglycone dehydration after hydrolysis (*m*/*z* 453.3374) (150 µM) observed after 30 min appeared to be transitory. At lower concentration (15µM), an aglycone-*O*-methylation (*m*/*z* 485.3625) after hydrolysis was observed after 30 min and slowly decreased over time, indicating the different partial metabolization between both concentrations (as previously observed for the degradation rate) (Figure 2a,b). Concerning UA-3-*O*-PP, Figure 2c,d shows a metabolite set formation over time at both concentrations. After 30 min, all the aglycone formed by hydrolysis appeared hydroxylated (*m*/*z* 471.3479) and further reduced (*m*/*z* 469.3324) for the next 30 min in parallel with a simple hydrolysis (*m*/*z* 453.3530), which was only also observed after 60 min at 150 µM. At this concentration, hydroxylation (*m*/*z* 603.4034) of the unhydrolyzed molecule first occurred rapidly, with further hydrolysis later (*m*/*z* 471.3479), while the proportion of hydroxylated and reduced aglycone (*m*/*z* 469.3324) also increased with time. The heatmap allows the visualization of the metabolic evolution over time, but further exact metabolite quantifications are necessary. The formation of the unmodified aglycone was only observed for the synthetic ester, which can be considered to some extent as a prodrug for this triterpenic acid (ursolic acid) which also possesses good antiparasitic activity [23]. The aglycone of the natural esters was proven to be much less effective than the esters [9] and was only detected as aglycone metabolites after further metabolization, but the activity of these metabolites need further evaluation. Active metabolites’ identification is also complementary for pharmacodynamic studies. Indeed, PTs display a large panel of pharmacological activities, suggesting a multi-target effect and a potential antiparasitic activity on different parasite life stages [35]. Currently, information on a precise mechanism of action for the tested triterpenic esters is lacking. Some UA derivatives have shown antiplasmodial activity on β-hematin formation or hemin degradation (via hydrogen peroxide or glutathione) [36], while antitrypanosomal activity of PTs can be explained by their known protein kinase C inhibition [37]. Further research is needed to assess if the triterpenic esters studied here are active on these targets or might have other mode(s) of action(s).

## 3. Materials and Methods

All used organic solvents (VWR, Leuven, Belgium) were HPLC grade. Water was purified and deionized with a Milli-Q system manufactured by Millipore (Bedford, MA, USA). The estradiol valerate (purity 99%) was purchased from Aca Pharma NV (Certa, Nazareth, Belgium) and used as internal standard. Procaine hydrochloride (purity >97%) and diclofenac sodium salt (≥98%) were purchased from Sigma-Aldrich (Saint Louis, MO, USA) and Fagron, Belgium, respectively. Pooled male mouse liver microsomes, NADPH regenerating system and 0.5 M potassium phosphate (pH = 7.4) were obtained from Corning (Fischer Scientific, Tournai, Belgium) while human plasma was obtained from CHU patients (O-, Liège, Belgique). HCl 37% (Acros Organics) was used to prepare pH 1.2 acidified water.

### 3.1. Compound Isolation and Synthesis

The mixture of 8TTE was isolated from the dichloromethane extract of *Keetia leucantha* twigs as described by Bero et al. [9] (Supplementary Materials Figure S5). UA-3-*O*-PP was hemi-synthetized as previously described from ursolic acid [11] (Supplementary Materials Figure S6). The compounds purity was assessed using an Accela HPLC-PDA system (Thermo Fisher Scientific Inc., Bremen, Germany) at 210 and 310 nm for hemi-synthetic (>97%) and natural (>98%) compounds, respectively as described below. Twigs of *Keetia leucantha* (K. Krause) Bridson (syn. Lectronia leucantha Krause, Rubiaceae) were collected in Benin (Adjarra, Ouémé) in July 2011 and August 2012 and identified at the National Botanic Garden of Belgium in Meise (in comparison to voucher number BR0000005087129).

### 3.2. Standard solutions

Methanolic calibration and validation standards were analyzed three times (n = 3) with three series of experiments (k = 3) at four concentration levels (m = 4) (100, 50, 10 and 5 μg/mL for calibration standards and 75, 32.5, 10 and 2.5 or 1.25 μg/mL for validation standards). For 8TTE, the pure mixture (>98% purity by HPLC-PDA) was used as standard. For plasma studies, six working solutions (m = 6) were prepared at 125, 100, 50, 10, 5 and 2.5 μg/mL in methanol and spiked (100 µL) in the blank biological matrix (200 µL) as recommended by EMA guidelines [38]. A suitable internal standard (IS), estradiol valerate, was added during working solutions preparation from a stock solution of 1 mg/mL in methanol to obtain a constant 100 μg/mL concentration. The six concentration levels (m = 6) of the calibration standards were analyzed in triplicate (n = 3) with 3 series of experiments (k = 3). Validation standards were prepared in the same way with four working solutions concentration levels (100, 50, 25 and 5 μg/mL). Final compound(s) concentrations in plasma were 0.8–41.6 µg/mL. Three independent samples (n = 3) at each concentration (m = 4) were analyzed in duplicate for 3 series of experiments (k = 3).

### 3.3. Plasma Samples

An amount of 1 mL acetonitrile was added to 300 µL samples that were submitted to 14,000 rpm centrifugation for 10 min at 4 °C (Eppendorf 5417R, Eppendorf AG, Hamburg, Germany). The supernatants were then evaporated with a CentriVap Benchtop Vacuum Concentrator, Labconco (Kansas City, MO, USA). The residues were dissolved in 100 μL methanol, vortex-mixed, sonicated 1 min on ice, vortex-mixed and filtered before HPLC-PDA analysis.

### 3.4. HPLC-PDA- and HPLC-PDA-HRMS Analysis

Samples were analyzed using an HPLC-PDA system consisting of a Thermo Accela pump, autosampler and photodiode array detector. The column used was a Poroshell 120 EC-C18, 100 × 4.6 mm packed with 2.7 µm particles. The flow rate was 0.8 mL/min using a gradient solvent system: solvent A (35%): H_2_O, solvent B (30%): acetonitrile, solvent C (35%): methanol, for 4 min, modified to reach solvent B (65%) and solvent C (35%) at 15 min, and then isocratic solvent system until 23 min. Starting conditions were restored from min 25 until min 30. The injection volume was 20 µL, and autosampler and column temperatures were set at 4 °C and 25 °C, respectively. Signals were monitored at 210 nm for hemi-synthetic C-3 ester, 280 nm for the internal standard and 310 nm for natural C-27 esters. For HPLC-PDA-HRMS analysis, samples were analyzed in the same conditions using an HPLC-PDA-HRMS system consisting of a Thermo Accela pump, autosampler, photodiode array detector and Thermo Scientific LTQ orbitrap XL mass spectrometer. High-resolution mass spectra were measured with the ESI source in the negative mode. The following inlet conditions were applied: capillary voltage −10 V (−31 V for estradiol valerate), tube lens −143.27 V (−118.27 V for estradiol valerate), ESI vaporizer temperature 275 °C, spray voltage 2.5 kV, sheath gas flow 15.00 a.u., auxiliary gas flow 15.00 a.u., sweep gas flow 15.00 a.u. Data acquisition and processing were performed with Xcalibur software. For metabolites analysis, raw data were converted into mzXML format with ProteoWizard [39] and then processed using XCMS software [40] into Worklow4Metabolomics 3.3 (W4M) [41]. CentWave was used for peak automatic detection and integration (peak picking, ppm = 10), while Camera was used for adducts and isotopes annotation [42]. Only peaks with twice the blank intensity were considered.

### 3.5. Method Validation

HPLC method was validated with three independent series of experiments. The same mobile phase was used all along with one series. Response function, linearity, selectivity, precision, trueness, accuracy, limit of detection (LOD) and limit of quantification (LOQ) and quantification range were investigated. The method selectivity was verified by HPLC-PDA-HRMS on the equipment as described above by checking mass spectra at the beginning, middle and end of the peaks. For quantification in plasma, HPLC-PDA chromatograms obtained after sample preparation from blank plasma, and spiked plasma were compared at the maximum absorption wavelength of 8TTE (310 nm), IS (281 nm) and UA-3-*O*-PP (210 nm) to confirm the lack of interference peak in blank plasma at compounds of interest retention times. The validation of the presented bioanalytical method agrees with EMA guidelines defined by trueness and precision values lower than 15% and expressed with the relative bias (RB) and the relative standard deviation (RSD), respectively [43]. In addition, total error (sum of systematic and random error) was used as decision criteria for the validation process [13,14,19,20,21,44]. Statistical analyzes were performed using JMP v12 software. The acceptance limits (λ) were set at ±20%, as is usually accepted for complex samples [19,45]. The probability of obtaining future results within these limits (β) was set at 95%.

### 3.6. Stability Tests

The compounds’ stability was evaluated by measuring their disappearance with the validated HPLC-PDA method. The protocol for microsomal stability was adapted from both mammalian microsomes manufacturer guidelines and literature [30,46]. Compounds were dissolved in DMSO (0.2% maximum final concentration) and tested at final concentrations of 15 µM and 150 µM. Triterpenic esters were incubated at different conditions at 37 °C, away from light over time (1 h: microsomes; 5 h: acidic condition; 24 h: plasma, aqueous and buffered solutions). Acidic stability was investigated by incubation with HCl acidified water at pH = 1.20 ± 0.02, assessed each day of the experiment by an Orion pH meter (Thermo Fisher Scientific Inc., Bremen, Germany). For microsomes stability, 1 mg/mL liver microsomes contained 100 mM potassium phosphate (pH 7.4) and a NADPH regenerating system (1.3 mM NADP^+^, 3.3 mM magnesium chloride and glucose-6-phosphate, 0.4 U/mL glucose-6-phosphate dehydrogenase). Each microsomal experiment was compared with negative control (without NADPH) for each compound, and the eventual loss was subtracted to exclude sources of compound loss other than microsomal metabolism. For plasma stability studies, entire plasma was used with a protein content of 74.2 mg/mL (calculated with Folin reagent using bovine serum plasma as standard) [47] (Supplementary Materials Figure S7). Plasma esterase efficiency was assessed by quantifying the disappearance of a known control compound (procaine) and its metabolite appearance (PABA) over a 24 h period (Supplementary Materials Figure S8). For plasma and microsomes samples at each time point, acetonitrile (the same volume as the sample) was used for enzymes’ inactivation or proteins’ precipitation as stop solution, spiked with estradiol valerate as internal standard (IS) to reduce variability (final analyzed concentration of IS = 280.5 µM = 100 µg/mL). The water samples buffered with 0.1 M potassium phosphate (pH 7.4) were also treated with this stop solution, whereas HCl acidified water samples were neutralized by a 0.1 M NaOH solution with IS.

### 3.7. Sampling and Data Analysis

All samples were centrifuged at 14,000 rpm for 10 min at 4 °C, and supernatants were evaporated under vacuum with a CentriVap (Labconco). The residue was recovered in methanol (VWR, HPLC purity) and injected in HPLC-PDA. AUCs were normalized with IS ones and results are expressed according to values at t = 0 (considered as 100% ester). For microsomes stability analysis, the remaining percentage versus the time was fit to a first decay function to estimate the rate constant of substrate loss (K_loss_) using the GraphPad Prism software 8.4.2 [48]. The liver microsomal in vitro *t*_1/2_, defined as the time required to reduce the concentration of a drug by 50%, was calculated assuming the elimination was of first-order kinetics [49]. The in vitro intrinsic clearance (C_int_) was calculated as µL min^−1^ mg^−1^ protein [50]. Regression parameters were calculated from calibration data using Microsoft Excel and GraphPad Prism 8 software. In vitro data are presented as the mean ± standard error and analyzed by GraphPad Prism 8 statistical software.

## 4. Conclusions

Despite the known pentacyclic triterpenoids antiparasitic potential, the first pass effect and the low bioavailability are limiting their further development and application as orally administrated drugs. Many efforts have been made to improve the defects with chemically modified derivatives, but only a few pieces of research have investigated the pharmacokinetic data of triterpenic esters, mostly showing higher in vitro efficacy and selectivity. In this study, a unique HPLC-PDA-HRMS method was validated for the quantification of both C-27 and C-3 triterpenic esters. This method was found to be selective, linear, accurate, true and precise in methanolic samples and plasma for both investigated esters types. Preliminary in vitro investigations on these esters showed plasma and pH 7.4 aqueous buffer stability, and also detected a certain degradation in a gastric environment and partial metabolism using mouse liver microsomes. Different hydrolysis mechanisms for the investigated esters were hypnotized. Metabolization was proposed to occur mainly via hydrolysis, hydroxylation, *O*-methylation and reduction, but additional studies are required. Specific formulations should be considered to increase the bioactive triterpenic esters bioavailability and stability. Further pharmacokinetic and permeability studies are thus needed, along with mode of action(s) investigations, to further assess the potential of these molecules.

## Figures and Tables

**Table 1:** Validation results obtained for the quantification method of UA-3-*O*-PP and 8TTE in MeOH.

Valid. Criteria		Concentration Levels (µg/mL) for Methanolic Samples								
		UA-3-*O*-PP				8TTE				
		2.5	10	37.5	100	1.25	2.5	37.5	75	100
Response function		Linear regression								
	Calib. range	4 points				5 points				
	µg/mL	2.5–100				1.25–100				
Trueness	Relative Bias (%)	−1.87	5.05	0.06	0.51	7.24	−2.80	−4.14	−2.38	−1.58
Precision	Repeatability (RSD ^1^ %)	2.18	1.66	1.72	1.19	4.78	4.72	4.55	2.17	4.08
	Intermediate precision (RSD ^1^ %)	3.84	2.88	2.71	2.46	4.41	4.16	3.81	2.99	4.23
Accuracy	(lower and uppertol. Limits %)	−15.59	−5.26	−8.34	−8.35	−3.27	−12.63	−13.23	−11.54	−12.18
		11.84	15.35	8.45	9.37	17.76	7.02	4.95	6.79	9.03
Linearity	Slope	1.003				0.982				
	Int.pt	0.122				−0.201				
	R^2^	0.999				0.997				

^1^ RSD: Relative Standard Deviation.

**Table 2:** Validation results obtained for the quantification method of UA-3-*O*-PP and 8TTE in spiked plasma.

Valid. Criteria		Concentration Levels ^1^ (µg/mL) for Plasma Samples							
		UA-3-*O*-PP				8TTE			
		5	25	50	100	5	25	50	100
Response function	Linear regression								
	Calib. range	5 points				6 points			
	µg/mL	5–125				2.5–125			
Trueness	Relative Bias (%)	−13.03	−0.57	−2.25	−0.57	15.47	7.18	−0.94	1.61
Precision	Repeatability (RSD ^2^ %)	2.89	2.70	1.57	1.79	1.81	2.66	1.05	1.52
	Intermediate precision (RSD ^2^ %)	2.65	3.25	3.19	3.72	1.48	2.47	2.49	1.83
Accuracy	(lower and uppertol. limits %)	−19.35	−9.67	−13.74	−13.97	11.85	1.28	−9.97	−3.26
		−6.71	8.52	9.23	12.83	19.09	13.09	8.07	6.48
Linearity	Slope	0.998				1.004			
	Int.pt	−0.551				0.755			
	R^2^	0.998				0.999			

^1^ Spiked Concentration; ^2^ RSD: Relative Standard Deviation.

**Table 3:** Validation results obtained for the quantification method of UA-3-*O*-PP and 8TTE in MeOH.

Concentration Level	Uncertainty (µg/mL)		Expanded Uncertainty (µg/mL)		Relative Expanded Uncertainty (%)	
(µg/mL)	UA-3-*O*-PP	8TTE	UA-3-*O*-PP	8TTE	UA-3-*O*-PP	8TTE
Methanolic sample method						
1.25	-	0.061	-	0.122	-	9.780
2.5	0.106	0.104	0.211	0.207	8.460	8.285
10	0.340	-	0.680	-	6.801	-
37.5	1.135	1.382	0.586	2.764	6.052	7.371
75	-	2.417	-	4.834	-	6.445
100	2.979	4.414	5.595	8.828	5.595	8.828
Plasma sample method						
5 ^1^	0.119	0.085	0.239	0.171	4.765	3.419
25 ^1^	0.879	0.686	1.759	1.373	7.034	5.491
50 ^1^	1.763	1.402	3.527	2.805	7.054	5.610
100 ^1^	4.186	2.022	8.371	4.045	8.371	4.045

^1^ Spiked Concentration.

**Table 4:** Elimination rate constant, half-life (min), in vitro intrinsic clearance (µL min^−1^ mg^−1^ protein) calculated for tested triterpenic esters and diclofenac.

	K_loss_ (min^−1^)	t_1/2_ (min)	Cl Int (µg/min/mg)
8TTE	0.009	76.82	18.04
UA-3-*O*-PP	0.003	264.30	5.24
Diclofenac	0.004	153.25	9.04

**Table 5:** Identification, prediction level and metabolic reactions involved of the detected metabolites.

**8TTE**	**Conc.**	**RT (sec)**	**Mol. Ion**	**Formula (M)**	***m/z*** **exp**	***m/z*** **thr**	**Δ** **ppm**	**ID vl**	**Reaction**
	150 µM	1005.73	[M − 1]^−^	C_30_H_46_O_3_	453.3361	453.3374	−2.8	2	Hydrolysis + dehydration
		884.76	[M − 1]^−^	C_30_H_46_O_4_	469.3323	469.3323	0	2	Hydrolysis + Reduction
	15 µM	884.76	[M − 1]^−^	C_30_H_46_O_4_	469.3323	469.3323	0	2	Hydrolysis + Reduction
		860.19	[M − 1]^−^	C_31_H_50_O_4_	485.3625	485.3631	−1.2	2	Hydrolysis + *0*-Methylation
**UA-3-** ***O*** **-PP**	150 µM	951.46	[M − 1]^−^	C_30_H_48_O_3_	455.3518	455.3530	2.6	2	Hydrolysis
		869.94	[M − 1]^−^	C_30_H_46_O_4_	469.3324	469.3323	0.2	2	Hydrolysis + Hydroxylation + Reduction
		717.12	[M − 1]^−^	C_30_H_48_O_4_	471.3479	471.3479	0	2	Hydrolysis + Hydroxylation
		983.24	[M − 1]^−^	C_39_H_56_O_5_	603.4034	603.4055	−3.5	2	Hydroxylation
	15 µM	951.46	[M − 1]^−^	C_30_H_48_O_3_	455.3518	455.3530	2.6	2	Hydrolysis
		869.94	[M − 1]^−^	C_30_H_46_O_4_	469.3324	469.3323	0.2	2	Hydrolysis + Hydroxylation + Reduction
		717.12	[M − 1]^−^	C_30_H_48_O_4_	471.3479	471.3479	0	2	Hydrolysis + Hydroxylation

## Supplementary Materials

The following are available online. Figure S1: HPLC-PDA chromatograms at different wavelengths of 8TTE, estradiol valerate (IS) and UA-3-*O*-PP analyzed by the validated method, Figure S2: Mass spectra of 8TTE triterpenic esters peaks (A–C) and UA-3-*O*-PP ester peak (D–F) taken at retention time corresponding to the beginning (A,D), the middle (B,E) and end (C,F) of triterpenic esters peaks. Mc = molecular ion ([M − 1]^−^) of natural C27 triterpenic esters with coumaric acid; Mf = molecular ion ([M − 1]^−^) of natural C27 triterpenic esters with ferulic acid; M = molecular ion ([M − 1]^−^) of hemi-synthetic C3 triterpenic ester, Figure S3: Accuracy profile of UA-3-*O*-PP in MeOH (A) and plasma (B) and of 8TTE in MeOH (C) and plasma (D) samples, respectively, with linear regression or after square root transformation. The steady line represents the relative bias, dashed lines indicate the β-expectation tolerance limits (β = 95%), and dotted lines represent the acceptance limits (±20%). The dots represent the relative back-calculated concentrations of the validation standards and are plotted according to their target concentration, Figure S4: Linearity profiles of UA-3-*O*-PP in MeOH (A) and plasma (B) and of 8TTE in MeOH (C) and plasma (D) samples, respectively. The dashed and dotted lined represent the β-expectations tolerance limits (β = 95%) and acceptance limits (±20%). The plane line is the identity line (y = x), Figure S5: Extraction and isolation process of the eight triterpenic esters mixture from the twigs of *Keetia leucantha*, Figure S6: Ursolic acid-3-*O*-phenylpropionate synthesis scheme, Figure S7: Schematic representation of Lowry assay, Figure S8: Plasma stability data on entire human plasma for procaine with PABA formation.

## Sample Availability

Samples of the compounds are not available from the authors.
