# Supplementary material for: A Validated HPLC-PDA-HRMS Method to Investigate the Biological Stability and Metabolism of Antiparasitic Triterpenic Esters
Source: Molecules. 2021 Nov 26;26(23):7154. doi: 10.3390/molecules26237154 (PMC8659078; doi:10.3390/molecules26237154)
Supplement: Supplementary file 1 [file molecules-26-07154-s001.zip › molecules-1457435-supplementary.pdf]

# Table of contents

1. HPLC-UV-PDA validated method ........ 3
2. Natural triterpenic esters extraction procedure ........ 7
3. Hemisynthetic triterpenic ester synthesis scheme ........ 8
4. Plasma protein content determination ........ 8
5. Plasma stability assay ........ 8
6. References ........ 9

# Figures Index

**Figure S1**. HPLC-PDA chromatograms at different wavelengths of 8TTE, estradiol valerate (IS) and UA-3-*O*-PP analyzed by the validated method. ........ 3

**Figure S2.** Mass spectra of 8TTE triterpenic esters peaks (A-C) and UA-3-*O*-PP ester peak (D-F) taken at retention time corresponding to the beginning (A,D), the middle (B, E) and end (C,F) of triterpenic esters peaks. Mc = molecular ion ($[M-1]^-$) of natural C27 triterpenic esters with coumaric acid ; Mf = molecular ion ($[M-1]^-$) of natural C27 triterpenic esters with ferulic acid; M = molecular ion ($[M-1]^-$) of hemi-synthetic C3 triterpenic ester. ........ 4

**Figure S3**. Accuracy profile of UA-3-*O*-PP in MeOH (A) and plasma (B) and of 8TTE in MeOH (C) and plasma (D) samples respectively with linear regression or after square root transformation. The steady line represents the relative bias, dashed lines indicate the β-expectation tolerance limits (β=95%) and dotted lines represent the acceptance limits (±20%). The dots represent the relative back-calculated concentrations of the validation standards and are plotted according to their target concentration. ........ 5

**Figure S4.** Linearity profiles of UA-3-*O*-PP in MeOH (A) and plasma (B) and of 8TTE in MeOH (C) and plasma (D) samples respectively. The dashed and dotted lined represent the β-expectations tolerance limits (β=95%) and acceptance limits (±20%). The plane line is the identity line (y=x). ........ 6

**Figure S5.** Extraction and isolation process of the eight triterpenic esters mixture from the twigs of *Keetia laucantha* ........ 7

**Figure S6.** Ursolic acid -3-*O*- phenylpropionate synthesis scheme ........ 8

**Figure S7.** Schematic representation of Lowry assay ........ 8

**Figure S8**. Plasma stability data on human entire plasma for procaine with PABA formation. ........ 9

# 1. HPLC-UV-PDA validated method

## 1.1 HPLC-PDA chromatogram

**Figure S1**. HPLC-PDA chromatograms at different wavelengths of 8TTE, estradiol valerate (IS) and UA-3-*O*-PP analyzed by the validated method.

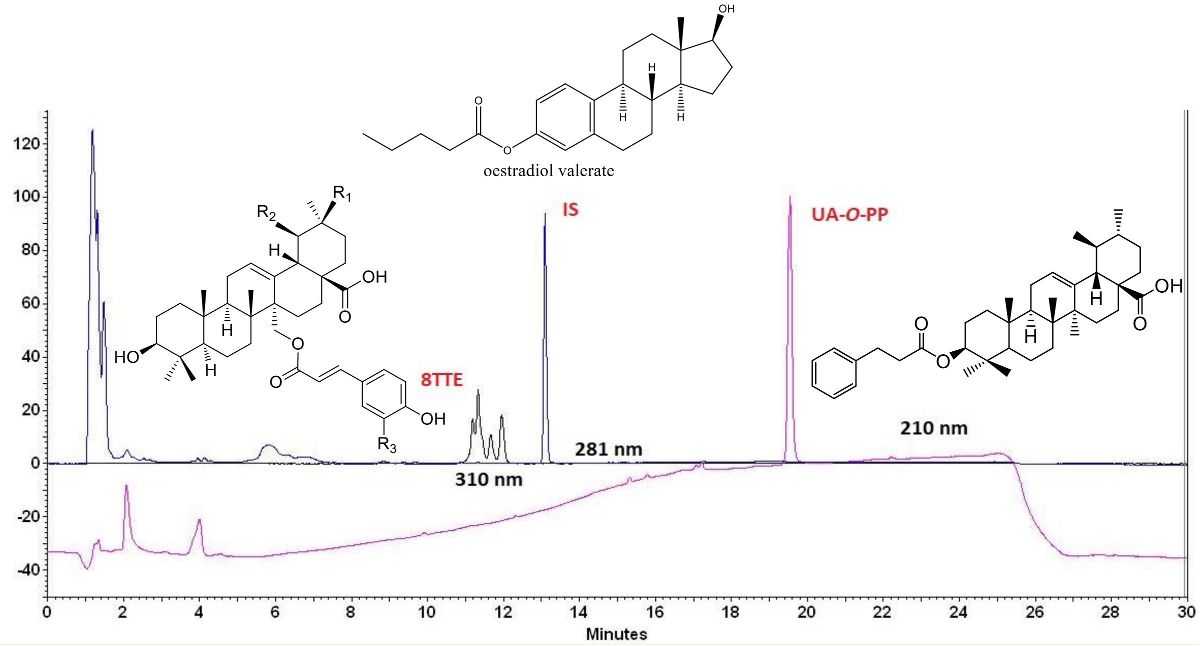

## 1.2 Mass spectra of triterpenic esters

**Figure S2.** Mass spectra of 8TTE triterpenic esters peaks (A-C) and UA-3-*O*-PP ester peak (D-F) taken at retention time corresponding to the beginning (A,D), the middle (B, E) and end (C,F) of triterpenic esters peaks. Mc = molecular ion ($[M-1]^-$) of natural C27 triterpenic esters with coumaric acid ; Mf = molecular ion ($[M-1]^-$) of natural C27 triterpenic esters with ferulic acid; M = molecular ion ($[M-1]^-$) of hemi-synthetic C3 triterpenic ester.

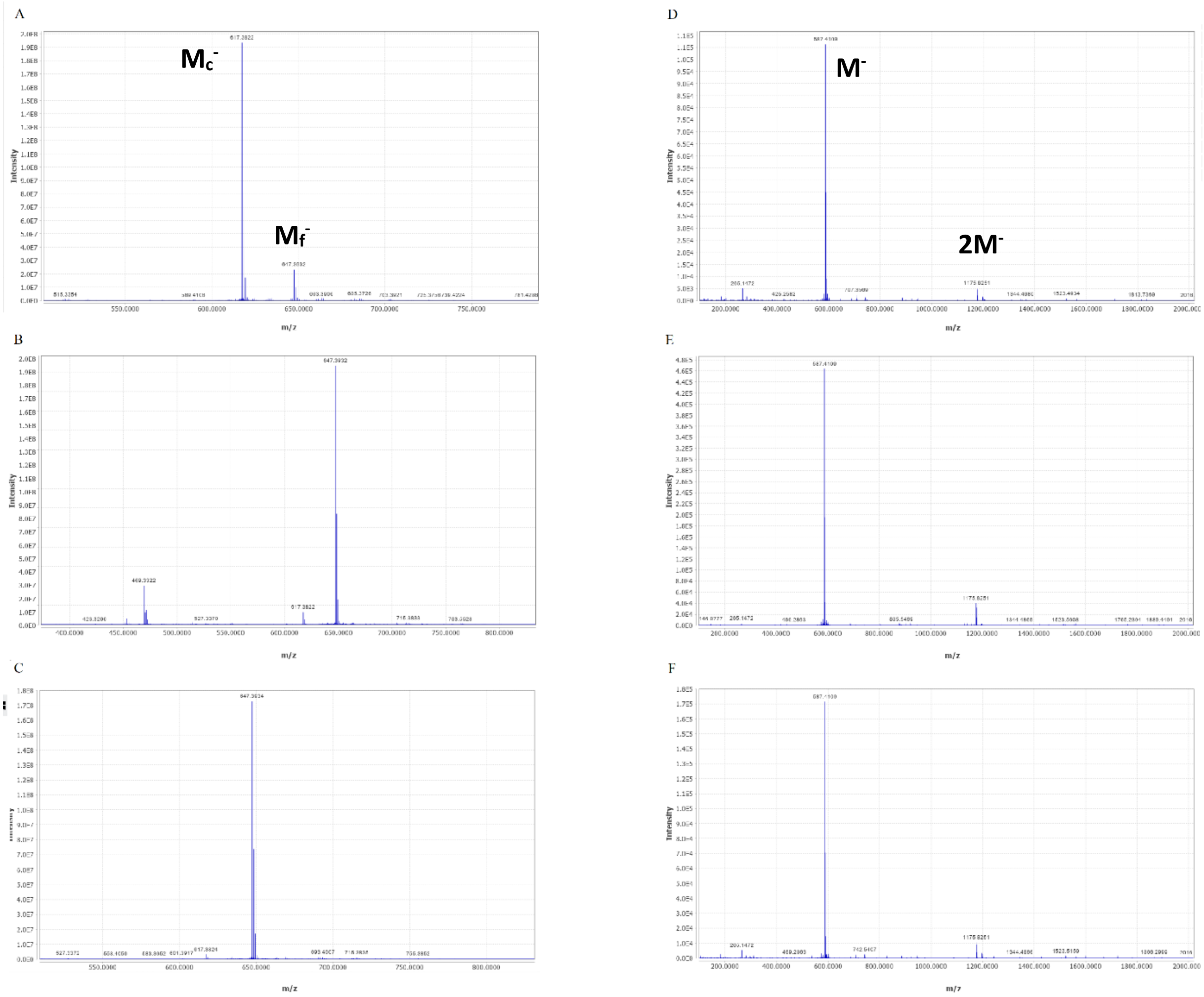

## 1.3 Accuracy profiles

**Figure S3**. Accuracy profile of UA-3-*O*-PP in MeOH (A) and plasma (B) and of 8TTE in MeOH (C) and plasma (D) samples respectively with linear regression or after square root transformation. The steady line represents the relative bias, dashed lines indicate the β-expectation tolerance limits (β=95%) and dotted lines represent the acceptance limits (±20%). The dots represent the relative back-calculated concentrations of the validation standards and are plotted according to their target concentration.

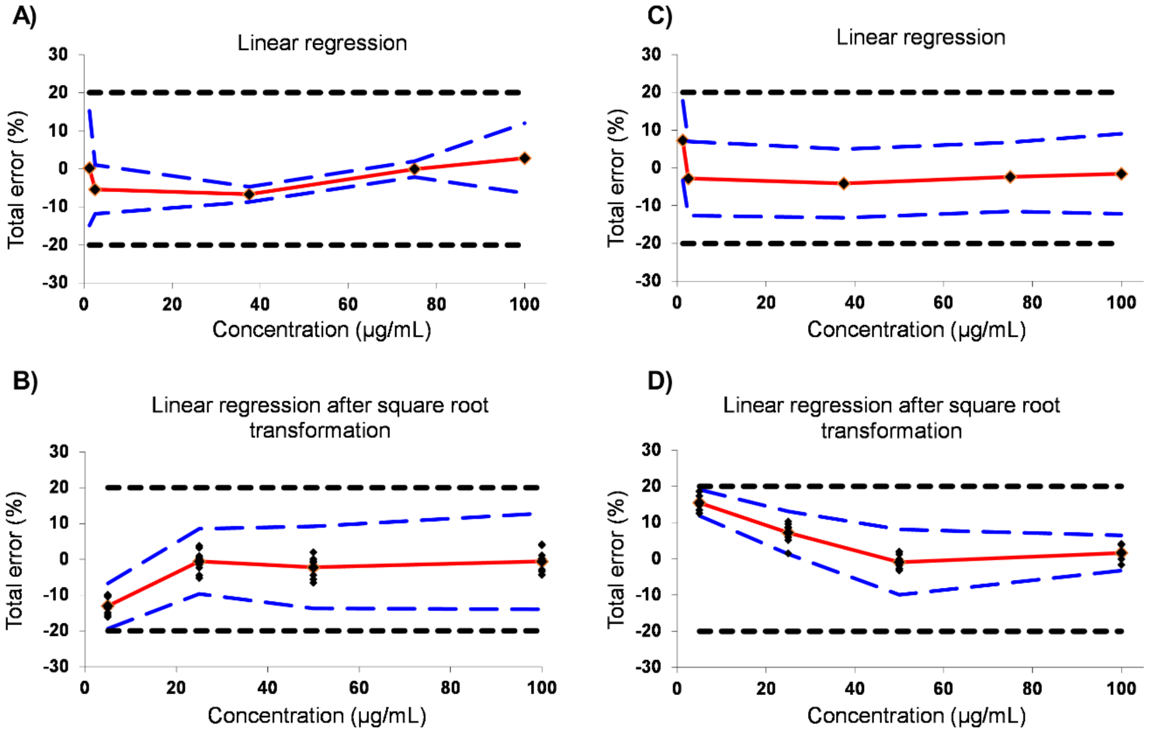

## 1.4 Linearity profiles

**Figure S4.** Linearity profiles of UA-3-*O*-PP in MeOH (A) and plasma (B) and of 8TTE in MeOH (C) and plasma (D) samples respectively. The dashed and dotted lined represent the β-expectations tolerance limits (β=95%) and acceptance limits (±20%). The plane line is the identity line (y=x).

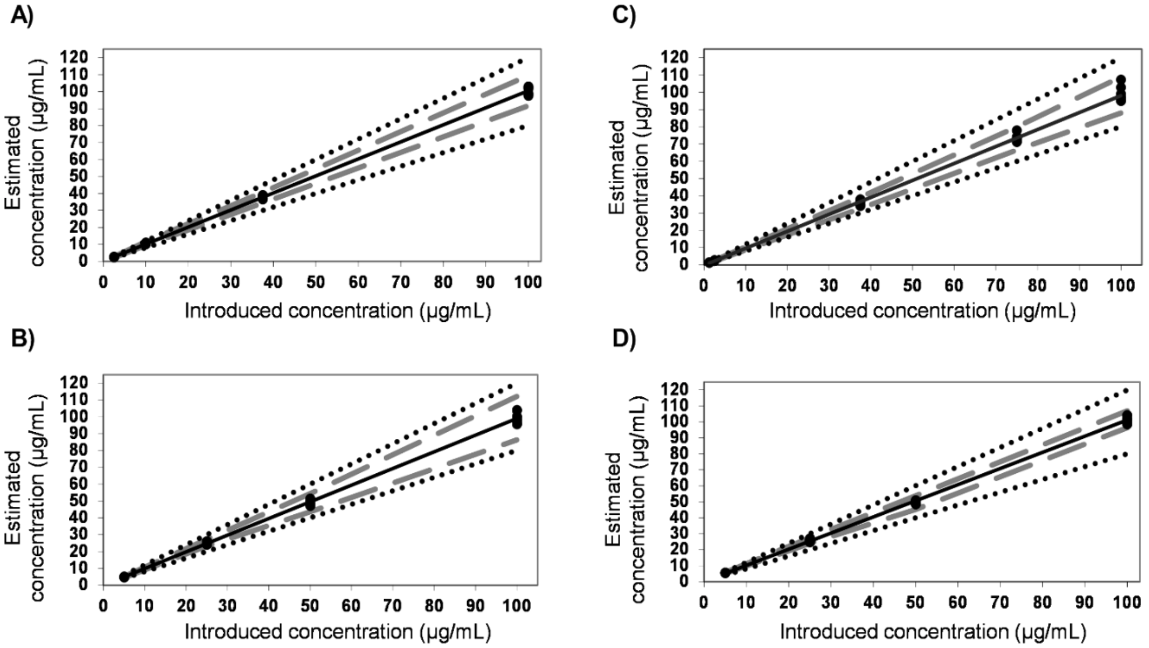

## 2. Natural triterpenic esters extraction procedure

**Figure S5.** Extraction and isolation process of the eight triterpenic esters mixture from the twigs of *Keetia laucantha*

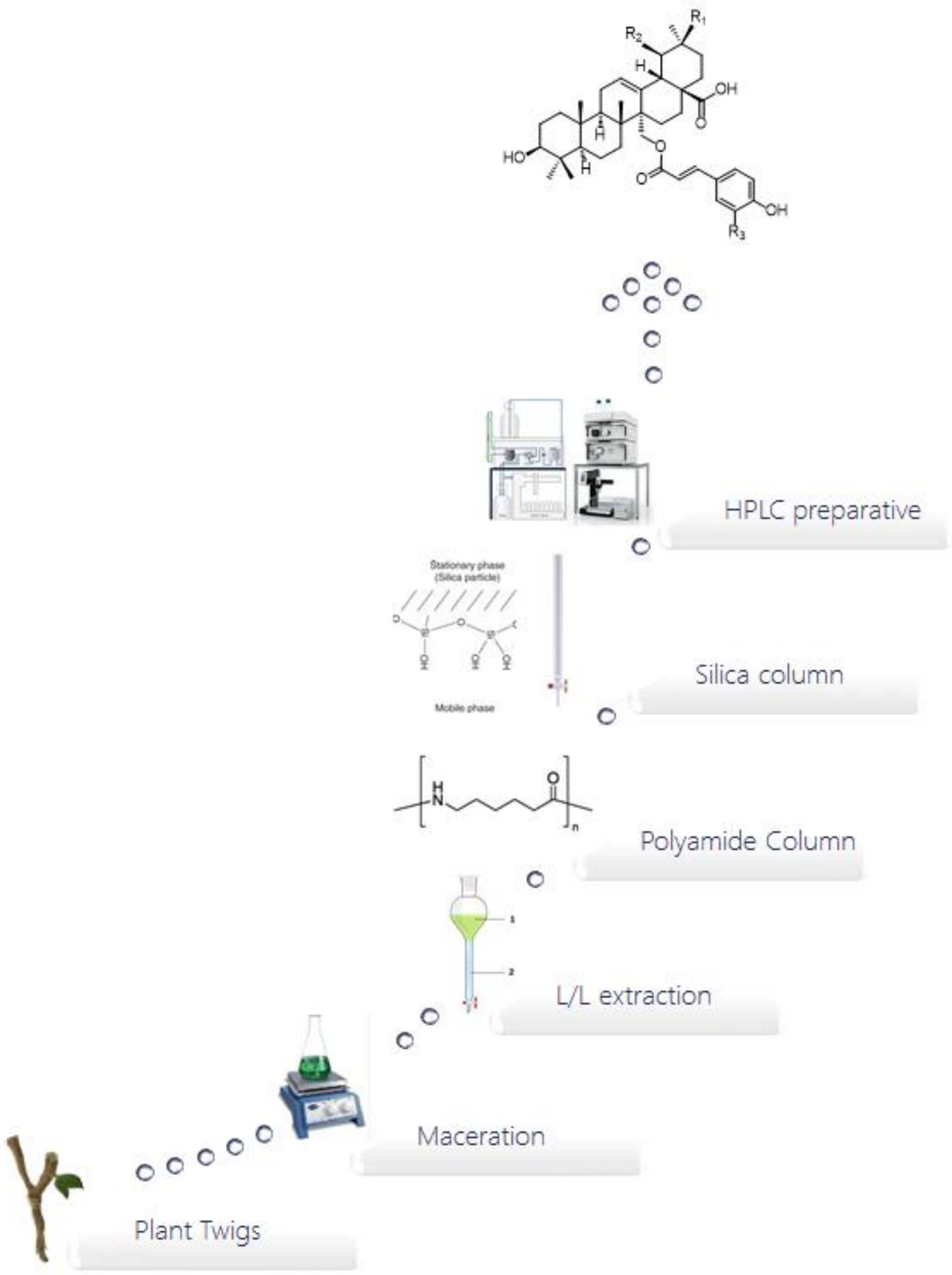


Isolation process described by Bero *et al.*

## 3. Hemisynthetic triterpenic ester synthesis scheme

**Figure S6.** Ursolic acid -3-*O*- phenylpropionate Synthesis Scheme

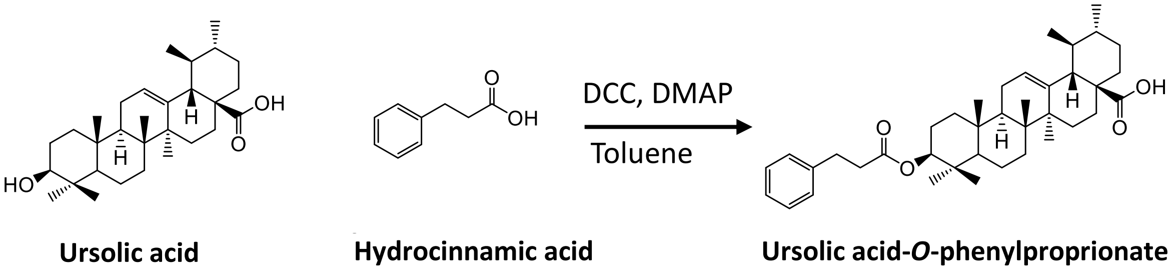


Synthesis is followed by a washing step with AcOEt/water with the organic phase dried over $MgSO_4$ and evaporated. The residue was redissolved and purified through a silica gel column as previously described.

## 4. Plasma protein content determination

The plasma protein dosage was done following the Lowry method (Figure S7) with a Biorad kit [1] with some ameliorations. Under alkaline conditions the divalent copper ion forms a complex with peptide bonds in which it is reduced to a monovalent ion. Monovalent copper ion and the radical groups of tyrosine, tryptophan, and cysteine react with Folin reagent to produce an unstable product that becomes reduced to molybdenum/tungsten blue. The color reaches 90% of its development within 15 min. Bovin serum plasma was used as standard for the dosage.

**Figure S7.** Schematic representation of Lowry assay

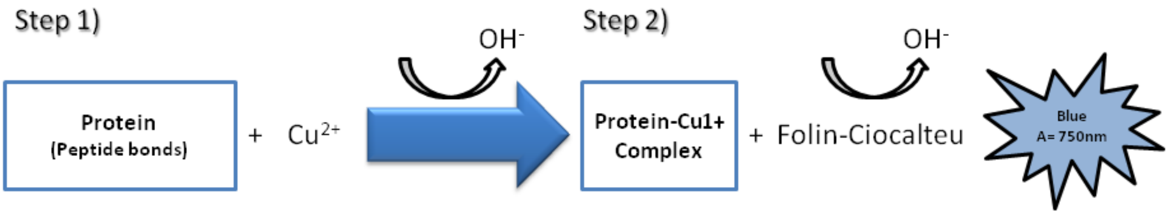


Source Scheme: Johnson M. et al [2]

## 5. Plasma stability assay

A control compound known to be metabolized by plasma  was also tested alongside each replicate of test compounds. Procaine was chosen as control as it is known to be quickly hydrolyzed by human plasma esterases (~10 min) into para-amino benzoic acid (PABA)[3]. The concentration of procaine or PABA produced in each sample was quantified by comparison of their peak areas in each injection to a standard curve produced using samples of known procaine or PABA concentrations. Concerning

human plasma enzymes stability for 24h, some literature data exist for analysis during 24h (last time-point of our analysis) with no loss of activity mentioned [4,5]. The disappearance of procaine up to 24h is shown in figure S8.

**Figure S8**. Plasma stability data on human entire plasma for Procaine with PABA formation.

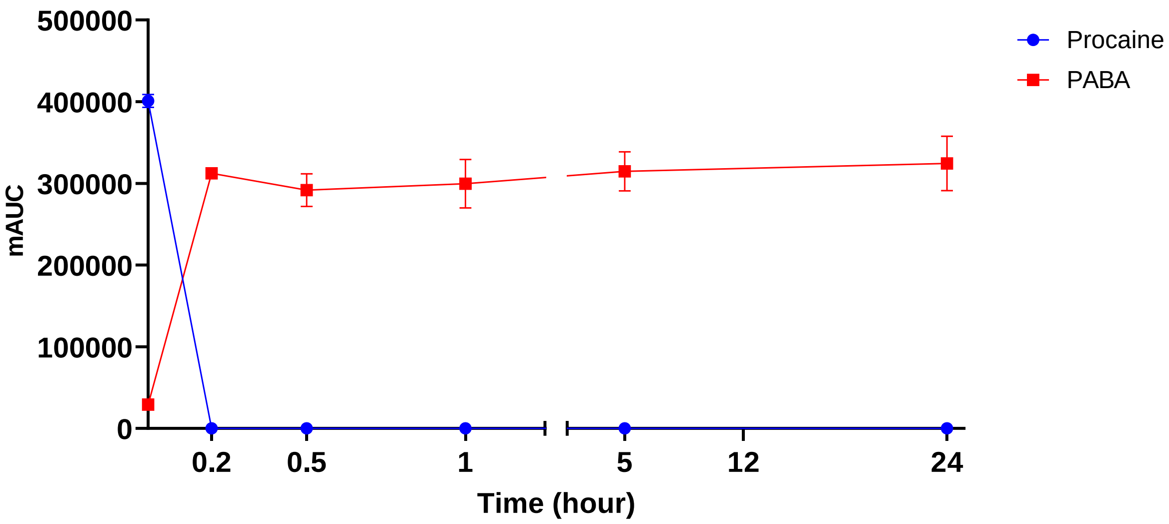


## 6. References


1. DC Protein Assay | Life Science Research | Bio-Rad. Available online: https://www.bio-rad.com/en-be/product/dc-protein-assay?ID=22faf97a-6b8d-4763-8b97-3dc530dcab66 (accessed on Apr 19, 2021).
2. Johnson, M. Protein Quantitation. *Mater. Methods* **2012**, *2*, doi:10.13070/mm.en.2.115.
3. Plasma Stability Assay. Available online: https://www.cyprotex.com/admepk/in-vitro-metabolism/plasma-stability (accessed on Nov 15, 2021).
4. Marinelli, L.; Fornasari, E.; Eusepi, P.; Ciulla, M.; Genovese, S.; Epifano, F.; Fiorito, S.; Turkez, H.; Örtücü, S.; Mingoia, M.; et al. Carvacrol prodrugs as novel antimicrobial agents. *Eur. J. Med. Chem.* **2019**, *178*, 515–529, doi:https://doi.org/10.1016/j.ejmech.2019.05.093.
5. Chayrov, R.; Parisis, N.A.; Chatziathanasiadou, M. V; Vrontaki, E.; Moschovou, K.; Melagraki, G.; Sbirkova-Dimitrova, H.; Shivachev, B.; Schmidtke, M.; Mitrev, Y.; et al. Synthetic Analogues of Aminoadamantane as Influenza Viral Inhibitors—In Vitro, In Silico and QSAR Studies. *Mol.* 2020, *25*.
